# Unveiling the dynamics of older person care: a qualitative exploration of the intersection between formal and informal caregiving from the perspectives of registered nurses in Greece

**DOI:** 10.1186/s12913-024-11401-5

**Published:** 2024-08-21

**Authors:** Lamprini Maria Xiarchi, Kristina Nässén, Lina Palmér, Fiona Cowdell, Elisabeth Lindberg

**Affiliations:** 1 Faculty of Caring Science, Work Life, and Social Welfare, Department of Caring Science, University of Borås, Borås, 501 90 Sweden; 2 School of Nursing and Midwifery, Birmingham City University, Westbourne Road, Birmingham, B15 3TN UK

**Keywords:** Holistic care, Nursing, Family caregiving, Older person care, Qualitative research

## Abstract

In the evolving landscape of older person care, the imperative to explore holistic approaches persists, especially in regions with distinct societal norms around informal caregiving. The deeply ingrained tradition of familial caregiving, often led by female family members, has historically compensated for healthcare system gaps in less integrated systems. Hence, societal expectations may affect registered nurses’ experiences and impact the quality of care for older persons. This study aims to describe the meaning of caring for older persons in care settings in Greece. Ten in-depth interviews with registered nurses in older person care settings underwent qualitative thematic analysis. Four themes emerged from the analysis: *(i) Living and bonding with older people as a basis for caring*,* (ii) Caring as a double-faced fulfillment*,* (iii) A sense of insufficiency in the caring relationship*, and *(iv) The encounter of existential issues creating loneliness.* This research provides insights into the meaning of caring for older persons, highlighting the experiences of the closest professional caregivers of older individuals. A specific capture of the entanglement of cultural norms, societal expectations, and their impact on professional caregivers’ experiences may contribute to quality care provision in systems rooted in familial caregiving traditions.

**Supplementary Information:**

The online version contains supplementary material available at 10.1186/s12913-024-11401-5.

## Background

In caring for older persons across diverse healthcare systems and transforming societies, there is a persistent need to extend further and explore holistic care supporting older individuals. In Greece, as in other Southern and Eastern European societies, the traditional model of familial caregiving has been deeply ingrained in the culture and social fabric, compensating for deficiencies in healthcare systems [1–4]. In these societies, familial caregiving has been strong, especially in regard to vulnerable individuals in care settings, and typically depends on kin, predominantly female family members [5, 6].

In Greece, as in the Mediterranean model, integration in health and social care for older people relies heavily on informal caregivers, although no official collaboration is provided [7, 8]. During previous years, the recession impacted the quality of health care, with understaffing and deficits in public health [9, 10]. A lack of nursing personnel, which created a disproportionate nurse‒patient ratio, has now been replaced by the presence of family members as informal caregivers in hospital and home care settings [5, 12]. At the same time, slightly more than 20% of the Greek population is over 65 years old and lives predominantly in rural areas, a percentage that is expected to increase by 2050 to be one of the highest in Europe [13]. Given the demographic situation, the ongoing need for quality care for older people is increasing, with a more persistent need in rural settings where there is less access to healthcare [11].

Familial caregiving appears necessary due to the system’s deficiencies in professional care for older individuals. Nevertheless, it is also culturally reproduced and has always been a part of society. Older persons often take on responsibilities as well, playing a significant role in the daily upbringing of their grandchildren, actively engaging in family gatherings, and providing financial support to their adult children [13]. Societal transformations, including urbanization, changes in family structures, and increased workforce participation among women, have led to shifts in the dynamics of older person care, putting a toll on the psychological well-being of informal caregivers [14]. In this scenario, registered nurses’ (RNs’) professional caring becomes important in safeguarding quality care for older persons, while systems may rely on and adapt to the needs of informal caregiving [5, 15].

The role of RNs stands as crucial in Greek society, carrying with it great social responsibility, imbuing RNs with ethical codes, but at the same time receiving low recognition [16, 17]. The influence of societal expectations on RNs’ conduct is rooted in historical connections to religion and ethics and is perceived as a societal role designating RNs as health educators or advocates responsible for patients in healthcare settings [16]. In addition, in contexts that rely on informal care, RNs are required to work closely to build trust while they bear significant responsibility for overall care [18]. Due to structural issues and unclear professional role definitions, nursing assistants in Greece are often seen as equivalent to RNs in similar roles [19]. Since RNs are responsible for all aspects of care and perform the same tasks as nursing assistants, there is a tendency for RNs to prioritize technical aspects, while physical and psychological support are still separated in Greek student nurses’ perceptions following the healthcare system’s medicalized view [20, 21]. Compared with technical aspects, listening to patients can be perceived as less important, such as observing the effects of medications and consulting doctors [22]. This might also be related to the high patient‒nurse ratio and autonomy of RNs, who are subject to a medically oriented culture that is typical of the Greek setting [23, 24]. Due to staff shortages and the existing culture, families’ trust in the healthcare system is low, reinforcing the perceived demand for informal caregiving as a complement to professional care [3].

Old age can lead to challenges, and it is crucial to understand the multidimensional aspects of how, for example, physical, mental, and existential aspects of health interplay and affect well-being in human life [25]. Caring for older persons demands a holistic approach in which the RN needs to preserve the patient’s perspective while navigating the complex interplay of cultural values, familial expectations, and healthcare demands. According to Husserl’s philosophy, an individual can be viewed as neither a part of an outer objective nor an inner subjective world but as part of a “humanly relational world” with many meanings [26]. In this view, social and historical structures are particularly important in revealing how the world is perceived [27].

Caring for older persons as RNs can be an experience with several intertwined meanings, and in societies where informal and formal caregiving intersect, extra attention is required to understand the experiences that are shaped within them. This study aimed to uncover the nuances of caring for older persons through the experiences of RNs working in environments strongly influenced by these longstanding societal caregiving norms.

### Study aim

The present study aims to describe the meaning of caring for older persons in care settings in Greece.

## Methods

The present study is steered by the theoretical principles of descriptive phenomenology [28] in the search for meanings of the phenomena that occur in our lifeworld. Lifeworld theory, originally introduced by Husserl [30], is harnessed to depict the lived experiences woven into our lifeworld. Husserl noted that individuals, in their daily lives, often assume a natural attitude, taking the world for granted without subjecting it to critical scrutiny. A phenomenon is characterized as an entity within the world encompassing both objective elements, such as tangible entities and observable events, and subjective aspects shaped by human experience. In this study, the phenomenon under investigation pertains to *caring for older persons in Greece*, from the scope of the experiences of RNs with a specific emphasis on the cultural context where caring takes place.

This process involves setting aside the researcher’s preconceptions and previous knowledge, allowing for the phenomenon to unveil its reflective aspect, which, according to Dahlberg et al. [28], is known as bridling. This is facilitated through discussions and reviews with coauthors throughout the research process.

### Context and care settings for older persons in Greece

In Greece, long-term care for older adults depends on a mix of formal and informal services, with limited availability of residential care facilities and home care provision within the healthcare system [29]. In this study, participants were selected from three diverse healthcare settings to provide varied experiences of older person care in Greece. These settings included residential care facilities, home care services, and a rehabilitation center that primarily engages with older persons with long-term conditions or in need of long-term rehabilitation. The care settings included in this study were partly financed by the older persons who received care and/or their families, except for the home care unit, where services were fully funded by the state.

### Participants

Ten RNs (eight women and two men) working in a rural area in northern Greece participated in the present study (Table 1). Two participants worked as RNs at a home care unit, four were RNs at a nursing home, and another four were part of a rehabilitation center. The RNs’ ages ranged from 27 to 55 years, and they were selected to participate in the study based on their ability to describe the phenomenon of “caring for older people (> 65 years) in Greece”. RNs’ experience in older person care ranged from 8 months to 17 years. According to convenience sampling, the participants in this study were selected based on their experience of the phenomenon, as well as their willingness to voluntarily participate in the study [31]. The RNs were not acquainted with or related to the authors before their participation in the study.

**Table 1:** Characteristics of the participants

Characteristic	*N* (%) or Mean (SD)
Gender	
*Male*	2 (20%)
*Female*	8 (80%)
Age	36.2
Workplace	
*Nursing home*	4 (40%)
*Rehabilitation center*	4 (40%)
*Home Care*	2 (20%)
Job Experience in older person care (years)	6.66

### Recruitment of participants/Data collection

Lifeworld in-depth interviews [28] involving ten participants were conducted. Initial interviews with four participants occurred in October 2022, followed by interviews with six more participants from January to March 2023. Recruitment involved email announcements and workplace calls for participation through gatekeepers in each organization (directors, managers, and public servants). The interviews were conducted in person at the participants’ workplaces, with the researcher’s discreet presence and, when possible, between shifts. One interview was conducted online via Zoom.

Each interview lasted between 20 and 50 min, and audio recordings were transcribed verbatim by the researcher (the interview guide developed for this study is presented as supplementary material). An initial question focused on the phenomenon was succeeded by open-ended questions aimed at enhancing the comprehension of the phenomenon. Follow-up questions such as “What do you mean?“, “Could you elaborate?“, and “Can you provide more details?” were included as examples.

### Data analysis

The analysis was carried out based on the in-depth descriptions gained from interviews with the participants. Drawing inspiration from Reflective Lifeworld Research [28], an open and reflective attitude was maintained throughout the whole analysis process, and this was achieved through constant movement between the parts and the whole of the dataset and a bridled attitude toward the phenomenon. The analysis was structured around the methodological principles of qualitative thematic analysis based on descriptive phenomenology [32]. The aim was to search for patterns of meanings in the lived experiences of participants and organize them into themes that eventually comprised a meaningful wholeness of the phenomenon. First, the interview texts were read several times, opting for a broad understanding of the dataset, following a search for novel and unique aspects of the phenomenon. Subsequently, the meanings were marked, and the variations among them were compared to assemble the highlighted meanings and organize them in patterns. These patterns were subjected to further scrutiny, guided by a reflective and open-minded exploration of the data. This process involved a continuous oscillation between the entire narrative in relation to its constituent elements. Finally, the discerned patterns were methodically structured into coherent themes. This was facilitated through in-depth discussions among the researchers that aimed at understanding meanings as derived directly from the lived experiences of the informants.

### Ethical considerations

This study is in line with the principles of the World Medical Association’s Declaration of Helsinki [33]. With respect to access to and recruitment of participants, permission was obtained from the key persons in charge of each workplace. Before every interview, participants were provided with detailed information on the research process and were informed that their participation should be voluntary, that participation could be terminated at any time and that research data would be collected with confidentiality. Written informed consent was obtained from all study participants. The data were coded and treated confidentially in the data analysis and presentation. The study was approved by the University of Ioannina Research Ethics Committee (ref. number: 4001/25–01–2021), the Swedish Ethical Authority (ref. number: 2023–02102–01), and the INNOVATEDIGNITY Ethical Scrutiny and Advisory Board.

## Results

**Fig. 1:**
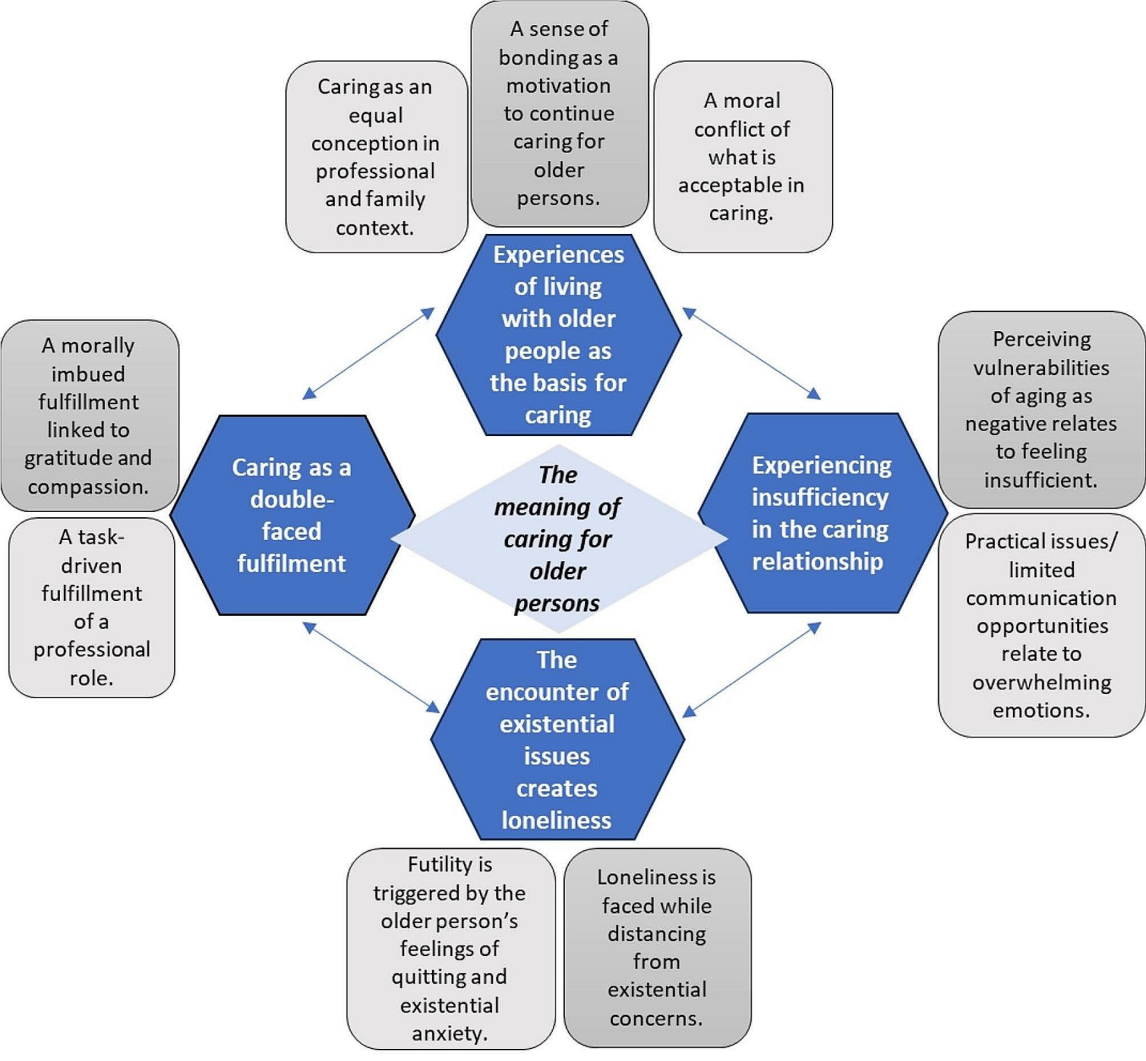
Overview of the main themes

The findings reveal the multifaceted phenomenon of caring for older persons based on the experiences of RNs. These revolve around the following main themes: (i) *Living and bonding with older people as a basis for caring*,* (ii) Caring as a double-faced fulfillment*,* (iii) A sense of insufficiency in the caring relationship*, and *(iv) The encounter of existential issues creating loneliness.* An overview of the main themes highlighting key points is provided below (Fig. 1).

### Theme 1: living and bonding with older people as a basis for caring

Exploring the phenomenon of caring for older persons based on the experiences of RNs addresses the relationship with the older person in terms of kinship. Balancing the maintenance of a professional identity with familiarity in the encounter with the older person expresses itself as a struggle where informal caregiving preconceptions and previous experience within the family act as primary references to professional caring. This indicates an unreflective perception of caring as an equal conception for both the professional and family contexts.

*When I came in here and did that*,* what came to me as an image was when our parents took care of us (…) That is*,* your parents help you*,* that is*,* in your first steps*,* in everything. So*,* when they get older*,* they want your help. It’s the… love*,* I’ll say it. (P.5)*

A sense of fulfillment is experienced through the perceived bonding that occurs in the caring relationships with older persons, and this relates back to the positive emotions deriving from mutually giving relationships within families and between generations. The caring relationship is experienced on a personal level, and familiarity enhances the sense of bonding in the relationship with older persons. Bonding emotions often serve as a motivation to continue caring for older persons professionally in the future.

*No. I could (change sector but…). No. (I wouldn’t change my job). Because I’m used to older people. I do not know. I cannot explain it to you. I don’t understand. However*,* I don’t want to change. I have learned to live with older people… A bond with older people! (P.5)*

The tight emotional bonds that accompany professional caring are expressed through examples of family caregiving. While RNs experience similar feelings of compassion and closeness in their relationships with their older family members, there is a lack of clear references to how professional caring evolves as a caring relationship. Experiences of caring within their family relate to parts of their professional identity and morality that imbue the profession in terms of enhancing social responsibility and compassion in their caring work.

*I simply treat a patient as if they were my own grandfather*,* my own grandmother so that I care for them the same way I would care for…Both my grandfather and my grandmother. … Because if my grandfather was to be in any place like here*,* I would want him to have the same (level of) care. (P.6)*

When the expectation of familial caregiving of older family members is not fulfilled (if the family is unable or unwilling to assist), this expands RNs’ responsibility as caregivers who are expected to compensate for missed care opportunities. In addition, normative expectations of how relatives should be involved in caring further enhance feelings of compassion for those who are unable to receive family care.

*It strikes me that they bring them here*,* and then they don’t care*,* not everyone…Their family…Very often. Not just those without children; those with children*,* too. Those who don’t have children have a reason*,* but those who do? (P.9)*

RNs who consider close care demanding might find it difficult to adjust to care settings such as nursing homes. More extensive experience in care settings is perceived as helpful in overcoming this difficulty since this entails a sense of security in terms of entering someone’s space. “Living” with older persons over a long period of close daily interaction creates an inner moral conflict concerning what is acceptable, as caring within the family is still the dominant frame of reference that guides the perceptions of what caring for older persons means.

*…When you are first introduced into this profession*,* in a Nursing Home*,* it is difficult. As the years go by*,* it gets easier*,* because that’s how you learn*,* you live with them*,* you will necessarily learn their peculiarities. It will be like having someone of your own in the house. (P.3)*

### Theme 2: Caring as a double-faced fulfillment

Caring for older persons involves emotions of self-fulfillment and a sense of professional accomplishment. The accomplishment of a professional role in terms of being good at one’s job is different from the self-fulfillment that comes from a moral obligation toward older persons reflected on a personal level.

Valuing a deeply expressed gratitude from older persons acts as the confirmation of the fulfillment of a moral obligation for RNs, as human beings who have chosen to engage in caring for vulnerable members of society.

*When someone is not able to eat*,* is not able to fulfill their basic needs and you are there to help them*,* in whichever way you can*,* this is pleasant to you. (P.6)*

Caring is experienced as deeply rewarding, and fulfillment arises from the gratitude and trust frequently expressed by older persons. “*Coming from the heart*” illustrates the personal elements that imbue the relationship with older persons and reflect fulfillment on an intimate level. Gratitude that is interpreted as *coming from the heart* is important as both a professional and personal reward for RNs, while it elevates RNs’ ethical role in doing good for older people.

*I feel happiness… the fact that they recognize that I am good at my job (…) The reward*,* the “thanks” that they are going to say*,* which I understand comes from their heart. (P. 1)*

Encountering vulnerability and complexity in caring and managing to overcome the thresholds of fragility, weakness, or dependency can lead to a deep sense of fulfillment. However, feelings of fulfillment are linked to the accomplishment of everyday tasks in caring, in which the caring relationship is described as the RN giving and the older person receiving, indicating a hierarchical positioning and normative preunderstanding of the older person as a passive receiver.

*…But especially in some cases*,* and at this age group that we have over here*,* you know that often their situation cannot be treated. And they look at you and you know they expect you to help them and even dress them; don’t you feel satisfaction when you dress someone up? At this point*,* you know they depend on you. Obviously*,* it’s even more important. They cannot serve themselves. (P.4)*

On the other hand, caring for individuals perceived as vulnerable enhances feelings of fulfillment, as this perception of vulnerability is also deeply connected to the possible reasons for choosing to care for older people professionally. The perceptions of dependency and fragility associated with old age evoke feelings of compassion, which contribute significantly to gaining satisfaction from caring through the fulfillment of an ethical obligation to care that is connected to the RNs’ professional identity. How their professional identity is colored in a moral sense can be exemplified in the expression *“Nursing chose me”.*

*…It’s nice when you realize that these people feel that you are “their own” person and they are really happy to see you and trust you (…) The truth is that nursing is not something I chose*,* it chose me (…) I am very lucky to have been here for so many years*,* despite the ups and downs over the years*,* I’m still here. (P.1)*

### Theme 3: a sense of insufficiency in the caring relationship

The caring relationship is viewed as an important part of caring where flexibility and individuality are valued as essential, in line with the principles of personalized care. It is stressed that the different needs of each older individual need to be respected to achieve optimized care.

*…Even (when caring) for the same person*,* I will try new methods*,* in different ways*,* in case I can do something extra to help this person*,* to help them… (P.6)*.

Efforts to prioritize and value the caring relationship coexist with practical concerns regarding task-based priorities in caring and limited time during shifts, which leaves little space for quality communication. This means “overwhelming” emotions from caring. A sense of inadequacy is enhanced by the perception of aging as a vulnerability that is negatively imbued, and this poses boundaries in the caring relationship. When meeting a patient’s reluctance to accept specific interventions, a feeling of insufficient care arises. Caring for older persons is followed by a sense of insufficiency, especially in regard to caring for individuals with cognitive conditions, e.g., dementia, or when having to establish communication with older persons who are hesitant to cooperate with caring, for example, in cases where an older person’s placement in a specific environment is not their own choice. Such a situation entails challenges for the RN when encountering the anxiety and grief experienced by the older person due to a situation with diminished hope and limited prospects.

*Some patients are very distant because of their situation; for example*,* with dementia*,* you cannot develop a relationship. (…) The older persons cannot understand… (P.2)*.

The entanglement of ethical values and professional identity is confirmed through disconnection in communication in caring, which is experienced as frustrating. In that case, an unpleasant sense of frustration prevails due to a failure to fulfill ethical goals, where the perception is that they are “not doing their job well”. Their sense of consistency and validity of professional identity is thus threatened.

*…Even though there are times when they (the older persons) make me angry*,* the way they act. (P. 9)*

### Theme 4: the encounter of existential issues creating loneliness

The inability to establish sufficient caring relationships is limiting and relates to feelings of futility. This becomes even more obvious when existential issues arise, usually through older individuals’ concerns about the end of life and upcoming death. At the end of life, existential pressure can result in older persons who might be unwilling to maintain relationships or continue living. Futility and a sense of inadequacy prevail, and this is usually triggered by the older person’s feelings of wanting to quit and the existential anxiety that can come with old age. Caring is then experienced as losing its purpose, and RNs thus confront insufficiency in their caring role.

When faced with older individuals’ existential concerns, RNs grapple not only with the limitations of their caregiving role but also with a profound sense of disconnection. This struggle, intertwined with feelings of inadequacy, appears to be a form of emotional distancing, where RNs become outside observers to overcome the emotional toll. Depersonalization can occur if individuals detach themselves as human beings, rationalizing or humorizing existential concerns. This is particularly obvious in caring situations where experiences of death are common.

*I usually try to calm them down (when thinking of death) … this is something rational that they are thinking. The fear they have… But nothing can be done. It is something acceptable*,* I usually tell them that even me might not be here tomorrow—laughs. Whatever the future will bring us is unknown… (P.2)*.

Being left with thoughts and having to deal with them alone contributes to feelings of loneliness. A sense of existential loneliness embodies a complex emotional isolation that relates to efforts to simultaneously navigate the existential struggles of older individuals while safeguarding their own emotional well-being and performing their professional responsibilities.

*I try to… when I go home*,* for example*,* I leave everything related to the nursing home behind*,* so that I don’t carry it with me and then it burdens me; in that sense*,* for example*,* because we have a lot of deaths here*,* most of them are very old. In the beginning*,* when I first got the job*,* I felt bad*,* it was painful… (P.3)*.

## Discussion

The findings of the present study unveil the meaning of caring for older persons, as reflected in the experiences of RNs in Greece, a context bound by strong social bonds and a great reliance on informal resources. The impact of cultural values has been studied extensively in relation to informal caregiving [34, 35]. At the same time, RNs collaborate with family, remaining at the forefront of caring for older individuals as the closest formal caregivers who can substantially contribute to the improvement of care and quality of life for older persons [36]. It is worth exploring this phenomenon, especially when it involves societies bound by values of familism and informal care.

Our findings highlight a deep sense of fulfillment tied to close caring relationships, and this in turn relates to RNs’ intention to continue their job in this field, which is a crucial finding in a sector with generally low retention rates and the need to attract more RNs [37, 38]. The experience of quality relationships in long-term settings has been shown to positively affect RNs despite adverse working conditions [39]. However, the shaping of professional identity in nursing, when influenced by informal resources and ethical responsibilities for the care of vulnerable individuals, places significant demands on the caring relationship. The intertwining of moral expectations from society with the system’s task-based demands results in confusion and inconsistency during nurse-older-person interactions. This contrasts with a holistic approach to caring and poses challenges and quality concerns in the caring experience, especially in a system characterized by limited resources and a predominantly task-oriented focus.

In the realm of caring, professionals are urged to embody both professionalism and moral integrity, allowing for the authentic expression of life in nurse‒patient interactions [40]. However, an unreflective normative hierarchy, where RNs are expected to lead and patients follow, contributes to RNs feeling insufficient and perceiving their caring role as demanding. The inability to reflect on these matters is also linked to nursing education and is a crucial matter for the well-being of those involved in caring [41]. This normative approach starkly clashes with the reality of older individuals, who grapple with existential vulnerability and suffering that cannot be effectively addressed through normative terms. The uniqueness of each person’s perspective must be acknowledged and integrated into the caregiving paradigm [42].

A lack of terminology derived from caring theory and a need for emphasis on holistic care within the system contribute to the challenges faced by RNs. From a caring science perspective, the importance of an open listening dialog is supported, as caring situations are full of meaning [43]. Our results show that the discrepancy between low institutional support for holistic care and aspects of close care that are perceived as overwhelming leads RNs to experience their caring role as extra demanding since they cannot conform to the system’s expectations regarding their role. The result delineates fulfillment as being recognized and confirmed as an RN in the relationship with the older person. This aspect contradicts the essence of a caring relationship and places expectations on the older person to be receptive and grateful. From a caring science perspective, fulfillment should ideally flow in the opposite direction, meaning that RNs’ self-fulfillment occurs when an older person’s unique situation is confirmed.

The emphasis on intergenerational family support in Greek society adds a significant layer to the caregiving dynamic [44]. The unreflective impact of cultural norms and values on kinship-like relationships in professional caring strengthens a sense of bonding with older individuals. This can form a basis for the authentic implementation of holistic approaches, putting the person in the center and benefiting both the carers and the older person in the long run. Such approaches align with the lifeworld perspective, which, among others, views well-being as moving into possibilities of engaging with others, while valuing the understanding of deeper existential horizons and the integration of these insights into care practices [45]. A lifeworld approach sees the meaning of caring as an interplay between the lifeworlds of the carers and the patients and this can represent a core element of RNs’ education [46]. This can contribute to a stronger sense of professional identity and ensure compassionate and holistic care. On the other hand, such a natural attitude toward caring imbued by specific cultural norms and values can further complicate the expectations and perspectives of professionals. RNs are loaded with complementing families in their assigned caregiving role as they become an important part of older individuals’ lives. This enhances the already strong presence of familism values in this context, influencing the shaping of RNs’ professional identities and providing implications for clinical practice; for example, RNs are tasked with an obligation to compensate for the absence of family care when family caregiving expectations are unmet and experience a sense of added responsibility. This research indicates the need for a framework concerning how RNs could support and work alongside family caregivers. According to Castro et al. [47], RNs need to recognize and support informal caregivers, as informal caregiving can cause distress for both the person in need of care and the informal caregiver. With all the good intentions of informal caregiving, it must always be acknowledged that it is mostly performed by untrained and unpaid family members, most often women of middle or old age.

Another important dimension relates to the fact that the spaces within which caring occurs are crucial for older people (e.g., nursing homes, home care, and rehabilitation). Personal-professional boundaries need to be considered, especially when the home becomes a place for caring, and in situations in which the older person’s quality of life tends to decline due to changes in the older person’s living space [48–50]. A person’s dignity depends, among other things, on the caregiver’s ability to create the appropriate space and time while being present [51]. However, this can prove to be a challenging task for RNs when caring for older persons, as spaces are redefined for older individuals and acquire new meanings on spatial, temporal, and emotional levels [52].

On the existential level, caring for older persons in their space of living addresses life in all its aspects, including loss and death. Existential pressure is common for both older individuals and RNs, as they confront an imminent death or tiredness of life from older individuals, which is difficult to address [53]. A loss of purpose and direction in caring impacts RNs, while challenging feelings tend to be avoided through depersonalization with a sense of detachment, which leads to loneliness for RNs. Loneliness can be viewed as a form of suffering that is experienced on a personal level and is referred to as an existential *suffering of life* [54]. Furthermore, depersonalization represents not only a coping mechanism but also a burnout dimension for RNs that has been previously found to be related to avoidance of grief in the encounter of patients’ deaths in long-term care settings [55]. A lack of support from the environment regarding existential matters possibly enhances loneliness and challenges in RNs’ jobs, which tend to remain unreflective.

### Strengths and limitations

A notable strength of the study is its exploration of the context of older person care in Greece. The study’s qualitative direction and focus on exploring phenomena within a certain context contribute to the understanding of the meaning of caring for older persons, especially in a setting marked by subsequent resource limitations. While these findings are crucial for similar healthcare systems and contexts, including informal caregiving resources for older persons, the focus on older person care in Greece may limit the transferability of the findings to other cultural contexts that might display different cultural factors and low reliance on informal caregiving. Future research could explore similar themes in diverse settings to provide a more comprehensive understanding of the challenges and rewards of caring for older persons.

In this study, interviews were conducted primarily in the workplaces of participating RNs, accommodating their schedules and workload responsibilities. Consequently, interview durations varied from 20 to 50 min, which may be considered a limitation as lifeworld in-depth interviews typically require longer periods to deeply explore experiences. Despite this, efforts were made to ensure that each interview allowed the phenomenon to emerge through the participants’ experiences. This was achieved within the available time by using follow-up questions and prompts to investigate each participant’s responses in depth.

All participants included in this study had sufficient work experience with older individuals in Greek older people care to be able to meaningfully describe the phenomenon. The fact that RNs’ professional experience in caring for older persons varied significantly *(from 3 months to 20 years)* can be considered a benefit, as including professionals at different stages of their careers adds to the diversity of experiences and promotes rich descriptions of the phenomenon. In terms of context, including experiences from novice and senior RNs provides a broad understanding of older person care in Greece among health professionals who have worked recently as well as before and during the recession years.

## Conclusions

In conclusion, this study provides valuable insights into the meaning of caring for older persons, capturing the interplay of societal expectations, cultural norms, and the challenges of a task-oriented yet partly informal older person care system. The identified aspects are key to a deeper understanding of the experiences of RNs in similar informally influenced healthcare systems and societies, offering implications for both clinical practice and further research in the field. The complexity of the context allows for the description of a multifaceted professional role. Additionally, this study provides significant insights into what impacts RNs’ professional identity which potentially influences their perspectives on their careers in this field. A discussion on what can reinforce RNs’ professional fulfillment is ongoing and this closely relates to integrating a holistic approach in caring. By recognizing and reflecting on the cultural norms and values that imbue RNs’ experiences, RNs can be better supported in their professional role. Developing frameworks that support RNs in working alongside family caregivers and incorporating holistic caring approaches into nursing education and practice are crucial steps toward achieving this goal. In this way, we can ensure that the care provided to older people is not only effective but also deeply respectful of their lived experiences and individual needs.

## Electronic supplementary material

Below is the link to the electronic supplementary material.

Supplementary Material 1

## Data Availability

The datasets generated and analyzed during the current study are not publicly available due to pending analyses for future publications. However, they are available from the corresponding author upon reasonable request.
