# Supplementary material for: Unveiling the dynamics of older person care: a qualitative exploration of the intersection between formal and informal caregiving from the perspectives of registered nurses in Greece
Source: BMC Health Serv Res. 2024 Aug 21;24:966. doi: 10.1186/s12913-024-11401-5 (PMC11340187; doi:10.1186/s12913-024-11401-5)
Supplement: Supplementary file 1 — Supplementary Material 1 [file 12913_2024_11401_MOESM1_ESM.docx]

**The phenomenon of *caring for older persons*- Interview Guide**

Work experience:

- How long have you been in the field of caring for older persons?
- How long have you been in this job?
- What is your educational background as a nurse?
- How come you chose to engage in caring for older persons?

Job responsibilities:

- Could you describe one typical day in your work?
- What does your work entail when it comes to caring for older persons?
- What are your responsibilities?

The meaning of caring for older persons:

- How is it to care for older persons?
- What does it mean for you to care for older persons as part of your job?
- Could you recall any aspects of caring that you consider important?
- Can you give me examples of caring for older people? / Is there any particular example that characterizes these aspects of caring?
- What does the relationship between you and older people entail? How do you view yourself within the nurse-patient relationship?
- As a nurse have you encountered any issues that are particularly relevant to gender? /Have any issues related to gender arised within the caring process?

Future perspectives:

- Do you see yourself continuing caring for older persons in the future?/ Would you continue working in caring for older persons?
- What would do you think is important in caring for older persons?
